# Supplementary material for: From sequence to enzyme mechanism using multi-label machine learning
Source: BMC Bioinformatics. 2014 May 19;15:150. doi: 10.1186/1471-2105-15-150 (PMC4229970; doi:10.1186/1471-2105-15-150)
Supplement: Additional file 2 — Java code of ml2db. Additional file ml2db_code.tar.gz contains the Java source code to run the multi-label machine learning experiments and save the results to database. The code’s Javadoc is included. [file 1471-2105-15-150-S2.zip › additional file 2/ml2db/ml2db/doc/main/ml2db/mulan/mulanarff/database/MulanArffInstancesLoader.html]

MulanArffInstancesLoader


JavaScript is disabled on your browser.


- Overview
- Package
- Class
- Use
- Tree
- Deprecated
- Index
- Help

- Prev Class
- Next Class

- Frames
- No Frames

- All Classes

- Summary:
- Nested |
- Field |
- Constr |
- Method

- Detail:
- Field |
- Constr |
- Method


main.ml2db.mulan.mulanarff.database

## Class MulanArffInstancesLoader

- java.lang.Object
- - main.ml2db.mulan.mulanarff.database.MulanArffInstancesLoader

- ---

    

  ```
  public class MulanArffInstancesLoader
  extends java.lang.Object
  ```

  Loads instances from file, given an arff database record identifier.

  Version:
  :   7 Jul 2011

  Author:
  :   Luna De Ferrari ldf(at)st-andrews.ac.uk

- - ### Constructor Summary

    Constructors

    Constructor and Description

    `MulanArffInstancesLoader()`
  - ### Method Summary

    Methods

    | Modifier and Type | Method and Description |
    | --- | --- |
    | `static mulan.data.MultiLabelInstances` | `generateDataSet(int arffRecordId, DbManagers dbMan)` |
    | `static mulan.data.MultiLabelInstances` | `generateDataSet(java.lang.String arffFilePath, java.lang.String xmlFilePath, DbManagers dbMan)` |
    | `static IdMultiLabelInstances` | `generateIdInstances(int arffRecordId, DbManagers dbMan)` |

    - ### Methods inherited from class java.lang.Object

      `equals, getClass, hashCode, notify, notifyAll, toString, wait, wait, wait`

- - ### Constructor Detail


    - #### MulanArffInstancesLoader

      ```
      public MulanArffInstancesLoader()
      ```
  - ### Method Detail


    - #### generateDataSet

      ```
      public static mulan.data.MultiLabelInstances generateDataSet(int arffRecordId,
                                                   DbManagers dbMan)
      ```


    - #### generateDataSet

      ```
      public static mulan.data.MultiLabelInstances generateDataSet(java.lang.String arffFilePath,
                                                   java.lang.String xmlFilePath,
                                                   DbManagers dbMan)
      ```


    - #### generateIdInstances

      ```
      public static IdMultiLabelInstances generateIdInstances(int arffRecordId,
                                              DbManagers dbMan)
      ```


- Overview
- Package
- Class
- Use
- Tree
- Deprecated
- Index
- Help

- Prev Class
- Next Class

- Frames
- No Frames

- All Classes

- Summary:
- Nested |
- Field |
- Constr |
- Method

- Detail:
- Field |
- Constr |
- Method
